# Correlation Between Hormonal Modulation and Pregnancy Outcomes: The Impact of Estrogen Priming and Endometrial Receptivity in Intrauterine Insemination

**DOI:** 10.7759/cureus.85345

**Published:** 2025-06-04

**Authors:** Hadia Riaz, Noor Fatima, Soobia Pathan, Marvi Memon, Abdul Ghafoor, Shama Chaudhry, M Khaliq

**Affiliations:** 1 Obstetrics and Gynecology, Dr. Ziauddin Hospital, Karachi, PAK; 2 Gynecology, Omar Medical Complex, Ghakhar Mandi, PAK; 3 Pharmacology and Therapeutics, Liaquat Institute of Medical and Health Sciences (LIMHS), Thatta, PAK; 4 Medicine, Bolan University of Medical and Health Sciences, Quetta, PAK; 5 Obstetrics and Gynecology, Ziauddin University, Karachi, PAK; 6 Pathology, University of Health Sciences, Lahore, PAK

**Keywords:** endometrial thickness, estradiol, estrogen priming, fertility, hormone modulation, iui, pregnancy outcome

## Abstract

Background: Intrauterine insemination (IUI) is one of the most commonly used assisted reproductive techniques for couples experiencing fertility issues. This study examined the difference in pregnancy rates between women who received estrogen priming before the ovulation trigger and those who did not.

Methods: This observational study was conducted at a fertility clinic and involved 80 women who underwent IUI, aged 24-38 years. The study spanned 12 months. The participants were divided into two groups: those who would receive estrogen priming (n = 40) and those who served as non-primed controls (n = 40). The sample size was calculated using OpenEpi 3.0.0 (Dean AG, Sullivan KM, Soe MM. OpenEpi: Open Source Epidemiologic Statistics for Public Health, Version. www.OpenEpi.com, updated 2013/04/06). Patients in the primed group were administered estradiol valerate (2 mg/day) from days 3 to 10 of the menstrual cycle. Endometrial characteristics, including thickness and pattern, and serum hormone levels were evaluated prior to ovulation triggering. Clinical pregnancy was confirmed via ultrasound. Data were analyzed using IBM SPSS Statistics for Windows, Version 26.0 (Released 2018; IBM Corp., Armonk, NY, US), with t-tests and chi-square tests applied. A p-value of 0.05 was considered statistically significant.

Results: Estrogen priming significantly improved endometrial thickness, with the primed group demonstrating a greater mean thickness compared to the control group (9.5 ± 1.3 mm vs. 7.6 ± 1.1 mm, p < 0.001). A positive correlation was observed between endometrial thickness and pregnancy outcomes (r = 0.41, p = 0.005).

Conclusion: The use of estrogen significantly enhanced endometrial growth and was associated with improved pregnancy outcomes following IUI. Administering estradiol as part of hormonal support may optimize endometrial receptivity and improve the efficacy of clinical fertility procedures.

## Introduction

Intrauterine insemination (IUI) is one of the most commonly used assisted reproductive techniques, particularly for couples experiencing unexplained infertility, mild male factor infertility, or ovulatory disorders [1]. IUI is a cost-effective and minimally invasive procedure; however, its clinical pregnancy rate remains relatively low, with reported rates ranging between 10% and 20% per cycle [2]. The success of IUI is influenced by several variables, such as maternal age, ovarian responsiveness, sperm quality, and, importantly, endometrial receptivity [3]. Endometrial receptivity is a key determinant of implantation success, as it reflects the capacity of the endometrium to support embryo adhesion and invasion during the optimal implantation window [4].

The hormonal milieu, particularly the roles of estrogen and progesterone, plays an important role not only in the development and maintenance of the endometrium but also in achieving successful conception [5]. Recent evidence suggests that estrogen priming may enhance endometrial development and optimize treatment outcomes in women undergoing IUI [6]. Administering exogenous estrogen during the early follicular phase, prior to ovulation induction, may create a more receptive endometrial environment, thereby increasing the likelihood of embryo implantation [7]. Laboratory studies demonstrate that estrogen promotes endometrial proliferation, cellular differentiation, and the expression of implantation markers. However, despite these promising findings, clinical data on the impact of estrogen priming on IUI remain limited and inconclusive. This underscores the need for further investigation into the correlation between hormonal modulation and pregnancy outcomes in IUI [8].

This study was designed to evaluate the efficacy of oral estrogen priming (2 mg/day), initiated during the early follicular phase, in enhancing endometrial development and improving pregnancy outcomes in women undergoing IUI. The primary hypothesis was that estrogen priming would lead to a significant increase in endometrial thickness and a higher prevalence of the triple-line endometrial pattern, ultimately resulting in an improved clinical pregnancy rate compared to women receiving standard ovulation stimulation without estrogen priming.

## Materials and methods

This study was conducted at the Department of Obstetrics and Gynecology, Dr. Ziauddin Hospital, Karachi, and the Department of Pathology, Federal Postgraduate Medical Institute (PGMI), Lahore, and involved 80 women aged 24-38 years over a 12-month period, from March 2023 to February 2024. Participants were randomly assigned using a simple random sampling technique into two equal groups (n = 40 each). The estradiol priming group received 2 mg of oral estradiol valerate daily from cycle day 3 through day 10, while the control group received standard treatment without estrogen priming. The sample size was calculated using OpenEpi 3.0.0 (Dean AG, Sullivan KM, Soe MM. OpenEpi: Open Source Epidemiologic Statistics for Public Health, Version. www.OpenEpi.com, updated 2013/04/06), with a significance level (alpha) of 0.05 and a power of 80%. The study was approved by the institutional ethics committee (reference no. 14/3/425ms) and conducted in accordance with the Declaration of Helsinki. Written informed consent was obtained from all participants prior to enrollment. Inclusion criteria were women aged 24-38 years with regular menstrual cycles (21-35 days), anti-Müllerian hormone (AMH) levels ≥ 1.0 ng/mL, and a normal endometrial appearance on ultrasound. Exclusion criteria included a diagnosis of polycystic ovary syndrome (PCOS), low ovarian follicle count, prior failed IUI attempts, structural uterine or endometrial abnormalities, endometriosis, or any underlying endocrine disorder.

All participants underwent the same controlled ovarian hyperstimulation (COH) protocol. Beginning on day 3 of the menstrual cycle, each subject received daily intramuscular injections of 300 IU of recombinant follicle-stimulating hormone (FSH). Follicular development was monitored via transvaginal ultrasound on days 7, 9, and 11. When the dominant follicle reached a diameter of 18-20 mm, ovulation was triggered with an intramuscular injection of 10,000 IU human chorionic gonadotropin (hCG) (Pregnyl®). Endometrial thickness was measured in the sagittal plane using transvaginal ultrasound on the day of ovulation trigger. In addition to thickness, the presence of a triple-line endometrial pattern, considered indicative of a receptive endometrium, was recorded. IUI was performed 24-36 hours after hCG administration using processed semen samples containing at least 5 × 10^6^ motile sperm. Clinical pregnancy was confirmed by measuring serum β-hCG levels 14 days after insemination.

Data were analyzed using IBM SPSS Statistics for Windows, Version 26.0 (Released 2018; IBM Corp., Armonk, NY, US). Continuous variables such as age, body mass index (BMI), and endometrial thickness were compared using the independent two-sample t-test, and results were expressed as mean ± standard deviation (SD). Categorical variables, including the presence of a triple-line endometrial pattern and clinical pregnancy rates, were reported as frequencies and percentages and analyzed using the chi-square test. A p-value of <0.05 was considered statistically significant.

## Results

A total of 80 participants were evaluated, with 40 women assigned to the estrogen priming group and the remaining 40 serving as controls. As both groups were demographically and clinically homogeneous, valid comparisons of treatment outcomes were feasible. Notably, the estrogen-primed group demonstrated a significant improvement in endometrial parameters and clinical pregnancy rates. The baseline characteristics of the study participants are summarized in Table 1.

**Table 1:** Baseline characteristics of the study participants Data were evaluated using independent t-tests and chi-square tests. BMI: body mass index, TSH: thyroid-stimulating hormone.

Parameter	Estrogen group (n = 40)	Control group (n = 40)	p-value	Test statistic (t-test)
Age (years)	30.2 ± 3.6	29.7 ± 3.9	0.46	0.74
BMI (kg/m²)	26.1 ± 2.9	25.8 ± 3.1	0.61	0.51
Baseline TSH (mIU/L)	2.6 ± 0.7	2.5 ± 0.8	0.58	0.56

There were no statistically significant differences in age, BMI, or baseline thyroid-stimulating hormone (TSH) levels between the estrogen priming and control groups. The similarity in baseline characteristics allowed for a robust comparison of outcomes between the two cohorts. Table 2 presents the measurements of endometrial thickness and the presence of a triple-line pattern on the day of ovulation triggering.

**Table 2:** Endometrial parameters on the day of ovulation trigger **Statistically significant at p < 0.01). The chi-square test was applied for the categorical comparison of the triple-line pattern; the t-test was used for the comparison of endometrial thickness.

Parameter	Estrogen group (n = 40)	Control group (n = 40)	p-value
Endometrial thickness (mm)	9.5 ± 1.3	7.6 ± 1.1	<0.001**
Triple-line pattern (%)	75% (30/40)	45% (18/40)	0.008**

Women in the estrogen-primed group demonstrated significantly greater endometrial thickness compared to those in the control group. As a result, a higher proportion of patients in the priming group exhibited a triple-line endometrial pattern, indicating enhanced endometrial receptivity. Table 3 presents a comparison of pregnancy outcomes between the two groups.

**Table 3:** Clinical pregnancy outcomes *Statistically significant at p < 0.05. Clinical pregnancy = presence of gestational sac on ultrasound; biochemical pregnancy = positive serum β-hCG without confirmed gestation on ultrasound. β-hCG: beta-human chorionic gonadotropin.

Outcome	Estrogen group (n = 40)	Control group (n = 40)	p-value	Test statistic (χ²)
Clinical pregnancy rate	28% (11/40)	12% (5/40)	0.039*	4.24
Biochemical pregnancy	10% (4/40)	5% (2/40)	0.39	0.74

The higher clinical pregnancy rate observed in the estrogen-primed group suggests a positive association between estrogen priming and improved reproductive outcomes. Although no significant difference was found in the biochemical pregnancy between the groups, the findings indicate that estrogen priming significantly enhanced endometrial receptivity. Notably, the clinical pregnancy rate was approximately doubled in IUI cycles with estrogen priming, highlighting how a simple yet effective intervention can substantially improve fertility outcomes.

## Discussion

This study examined the relationship between estrogen priming, endometrial receptivity, and pregnancy outcomes in women undergoing IUI. Estrogen priming demonstrated a statistically significant effect on endometrial thickness, the presence of triple-line endometrial pattern, and clinical pregnancy rates compared to the control group. These findings underscore the critical role of estrogen in optimizing the endometrial environment for successful embryo implantation. Estrogen was shown to be essential during the follicular phase, enhancing endometrial development through improved vascularization, epithelial proliferation, and tissue growth, all of which contribute to increased endometrial receptivity. These results were consistent with previous research, suggesting that an endometrial thickness greater than 9 mm is associated with a higher likelihood of implantation and pregnancy [9]. Furthermore, the presence of a triple-line pattern on ultrasound served as a marker of uterine receptivity. Mounting evidence has proven that conducive implantation and pregnancy likely occur when the implantation window aligns with a receptive endometrium [10,11].

The administration of estrogen extended the receptive phase of the endometrium and promoted better synchronization between the embryo and the endometrial environment. Clinical pregnancy occurred in 11 women (28%) in the estrogen-primed group, significantly higher than in the control group, where only 5 women (12%) conceived (p = 0.039). This notable difference supports the therapeutic role of exogenous estrogen, particularly in women with thin endometrium or suboptimal endometrial response during assisted reproductive technologies [12,13]. Follicular development was comparable in both groups, suggesting that estrogen’s beneficial effect was localized to the endometrium and largely independent of ovarian function. No adverse effects were reported, indicating that low-dose estradiol is generally safe and well-tolerated. These findings are consistent with previous studies that demonstrated improved implantation and pregnancy outcomes following estrogen administration in IVF and intracytoplasmic sperm injection (ICSI) cycles [14,15]. However, the variability in dosing and administration protocols across studies underscores the need for standardization to optimize outcomes [16]. A key strength of this study lies in its randomized design, consistent stimulation protocol, and the use of objective measurements of endometrial parameters and clinical outcomes.

However, some limitations should be acknowledged. Although the sample size was adequate for the analysis, the luteal phase was not comprehensively evaluated. Additionally, while serum estradiol levels were monitored, this study did not assess specific endometrial receptivity markers such as integrins or HOXA10. Future research should explore the molecular aspects of endometrial receptivity and incorporate longitudinal tracking of patient responses to fertility treatments. Further refinement of estrogen priming protocols is also warranted to determine individualized hormonal regimens that align with a woman's physiological profile and reproductive potential, as suggested in previous literature.

## Conclusions

In conclusion, estrogen priming demonstrated significant benefits for women with low ovarian reserve undergoing IUI treatments. The addition of estrogen contributed to enhanced follicular development and improved endometrial receptivity, ultimately leading to better pregnancy outcomes. These findings support the use of estrogen priming as a promising adjunct in assisted reproductive protocols, with the potential to optimize cycle timing and increase the likelihood of successful implantation. However, clinical decision-making should be individualized, taking into account each patient's medical history and reproductive profile.

Future studies should concentrate on optimizing the timing, dosage, and duration of estrogen priming to maximize clinical effectiveness while minimizing associated risks. Comparative studies with alternative stimulation protocols could help clarify the relative advantages of estrogen priming. Moreover, an understanding of the molecular mechanisms underpinning its effectiveness may open new paths for more targeted and personalized fertility treatments.
